# Luteolin-Rich Extract of *Thespesia garckeana* F. Hoffm. (Snot Apple) Contains Potential Drug-Like Candidates and Modulates Glycemic and Oxidoinflammatory Aberrations in Experimental Animals

**DOI:** 10.1155/2022/1215097

**Published:** 2022-07-30

**Authors:** Uchenna Blessing Alozieuwa, Bashir Lawal, Saidu Sani, Amos Sunday Onikanni, Obinna Osuji, Yunusa Olatunji Ibrahim, Shukurat Bisola Babalola, Gomaa Mostafa-Hedeab, Abdulrahman A. Alsayegh, Sarah Albogami, Gaber El-Saber Batiha, Alexander T. H. Wu, Hsu-Shan Huang, Carlos Adam Conte-Junior

**Affiliations:** ^1^Department of Biochemistry, Faculty of Natural and Applied Sciences, Veritas University Abuja, FCT-Abuja, Nigeria; ^2^PhD Program for Cancer Molecular Biology and Drug Discovery, College of Medical Science and Technology, Taipei Medical University, Taipei and Academia Sinica, Taipei 11031, Taiwan; ^3^Graduate Institute for Cancer Biology & Drug Discovery, College of Medical Science and Technology, Taipei Medical University, Taipei 11031, Taiwan; ^4^Department of Biochemistry and Molecular Biology, Faculty of Science, Federal University Ndufu-Alike Ikwo, P.M., B 1010 Abakaliki, Ebonyi State., Nigeria; ^5^Department of Chemical Sciences, Biochemistry Unit, Afe Babalola University, Ado-Ekiti, Ekiti State, Nigeria; ^6^College of Medicine, Graduate Institute of Biomedical Science, China Medical University, Taiwan; ^7^Department of Chemistry, Faculty of Physical Sciences, Alex-Ekwueme Federal University Ndufu-Alike, Ebonyi State, Nigeria; ^8^Department of Biochemistry, Federal University of Technology, Minna, Nigeria; ^9^Department of Chemistry, Federal University of Technology, Minna, Nigeria; ^10^Pharmacology Department & Health Research Unit, Medical College Jouf University, Jouf, Saudi Arabia; ^11^Pharmacology Department Faculty of Medicine, Beni-Suef University, Egypt; ^12^Clinical Nutrition Department, Applied Medical Sciences College, Jazan University, Jazan 82817, Saudi Arabia; ^13^Department of Biotechnology, College of Science, Taif University, P.O. Box 11099, Taif 21944, Saudi Arabia; ^14^Department of Pharmacology and Therapeutics, Faculty of Veterinary Medicine, Damanhour University, Damanhour 22511, AlBeheira, Egypt; ^15^TMU Research Center of Cancer Translational Medicine, Taipei Medical University, Taipei 11031, Taiwan; ^16^The PhD Program of Translational Medicine, College of Medical Science and Technology, Taipei Medical University, Taipei 11031, Taiwan; ^17^Clinical Research Center, Taipei Medical University Hospital, Taipei Medical University, Taipei 11031, Taiwan; ^18^Graduate Institute of Medical Sciences, National Defense Medical Center, Taipei 11490, Taiwan; ^19^School of Pharmacy, National Defense Medical Center, Taipei 11490, Taiwan; ^20^PhD Program in Biotechnology Research and Development College of Pharmacy, Taipei Medical University, Taipei 11031, Taiwan; ^21^Center for Food Analysis (NAL), Technological Development Support Laboratory (LADETEC), Federal University of Rio de Janeiro (UFRJ), Cidade Universitária, Rio de Janeiro 21941-598, Brazil

## Abstract

The present study evaluated the polyphenolic contents and hypoglycemic, antioxidant, and anti-inflammatory effects of the diethyl ether fraction of *Thespesia garckeana* using various *in vitro* and *in vivo* models. Total phenol and flavonoid contents of the extract were 613.65 ± 2.38 and 152.83 ± 1.56 mg/100 g dry weight, respectively. The extract exhibited *in vitro* antioxidant activities against DPPH, FRAP, LPO, and ABTS with respective half-maximal inhibitory concentration (IC_50_) values of 30.91 ± 0.23, 16.81 ± 0.51, 41.29 ± 1.82, and 42.39 ± 2.24 *μ*g/mL. *In vitro* anti-inflammatory studies using membrane stabilization, protein denaturation, and proteinase activities revealed the effectiveness of the extract with respective IC_50_ values of 54.45 ± 2.89, 93.62 ± 3.04, and 56.60 ± 2.34 *μ*g/mL, while *in vitro* hypoglycemic analysis of the extract revealed inhibition of *α*-amylase (IC_50_64.59 ± 3.29 *μ*g/mL) and enhancement of glucose uptake by yeast cells. Interestingly, the extract demonstrated *in vivo* hypoglycemic and anti-inflammatory effects in streptozotocin- (STZ-) induced diabetic and xylene-induced ear swelling models, respectively. In addition, the extract improved insulin secretion, attenuated pancreatic tissue distortion and oxidative stress, and increased the activities of superoxide dismutase (SOD), catalase, and reduced glutathione (GSH), while reducing the concentration of LPO in the diabetic rats. A high-performance liquid chromatography (HPLC) analysis identified the presence of catechin (6.81*e* − 1 ppm), rutin (8.46 *e* − 1 ppm), myricetin, apigenin (4.019 *e* − 1 ppm), and luteolin (15.09 ppm) with respective retention times (RTs) of 13.64, 24.269, 27.781, 29.58, and 32.23 min, and these were subjected to a pharmacoinformatics analysis, which revealed their drug-likeness and good pharmacokinetic properties. A docking analysis hinted at the potential of luteolin, the most abundant compound in the extract, for targeting glucose-metabolizing enzymes. Thus, the present study provides preclinical insights into the bioactive constituents of *T. garckeana*, its antioxidant and anti-inflammatory effects, and its potential for the treatment of diabetes.

## 1. Introduction

Diabetes mellitus (DM) is a category of metabolic disorders that affect glucose, lipid, and protein metabolism and consequently affect the overall health status [1, 2]. The prevalence of diabetes is very high, affecting millions of people globally [3]. It is characterized by the development of insulin resistance, abnormal insulin signaling, oxidative stress [4], inflammation [5], and organ dysfunction [6], leading to decreased life quality and high mortality [7]. In the past few decades, DM, particularly type 2 DM (T2DM), has become a global health problem that threatens millions of people in both developed and developing countries [2]. According to the Diabetes Atlas (10th edition) of the International Diabetes Federation (IDF) [8], there are 537 million people living with diabetes in 2021, and about 783 million cases are expected by 2045 [8], compared to 151 million sufferers in 2000 [9–11].

Although the pathogenesis and pathophysiology of T2DM are extremely complex and not fully understood, accumulating evidence has revealed that free radical generation, oxidative stress, and inflammation play critical implicative roles in the development of T2DM [12, 13]. Inflammation, a complex physiological response to injury and infection, plays a pivotal role in the development of chronic disorders, including arthritis, asthma, atherosclerosis, and cancers [14]. Experimental and clinical studies have provided evidence of the important roles of inflammation in DM [15, 16]. Reactive nitrogen species (RNS) and reactive oxygen species (ROS) were clearly implicated in the pathology of various diseases including aging, cancers, cardiovascular diseases (CVD), neuronal impairment, and diabetic complications [14, 17]. During hyperglycemia, the generation of free radicals (RNS and ROS), autooxidation of glucose, and depletion of endogenous antioxidants lead to oxidative stress and inflammatory conditions, apoptosis of pancreatic islet *β* cells, and impaired insulin secretion [18]. In addition, inflammation and oxidative stress also contribute to the development of diabetic complications such as hypertension, retinopathies, nephropathies, and neuropathies [19, 20].

Over the years, synthetic and chemical antioxidants and antidiabetic medications have been developed for treating diabetes and its associated complications [21]. However, their clinical applications have been limited by the loss of efficacy and more importantly by associated side effects including diarrhea, lactic acidosis, flatulence, and acute hepatitis [22, 23]. Hence, there is a need to focus on seeking new alternatives. Moreover, evidence from traditional health practice and experimental studies suggested that natural products, particularly medicinal plants, are rich sources of therapeutic agents that can offer better efficacy with minimal side effects compared to conventional therapies [24–26]. Thus, exploring bioactive metabolites from medicinal plants may offer multieffect glycemic control while modulating oxidoinflammatory aberrations and improving prognoses of diabetes and its complications.

*Thespesia garckeana* F. Hoffm., known as ‘snot apple' or ‘kola of Tula,' is a reputable medicinal plant that is widely distributed in Africa and some other tropical countries [27, 28]. As a traditional medicine, the plant is widely used for treating rheumatism, infections, diabetes, liver diseases, reproductive impairment, and other diseases [28, 29]. Several biological activities of the plant were also reported in the literature [28, 30–32]. Herein, we evaluated the hypoglycemic, antioxidant, and anti-inflammatory effects of the diethyl ether fraction of *T. garckeana* using various *in vitro* and *in vivo* models. Further characterization of the extract revealed the presence of various compounds which were subjected to a pharmacoinformatics analysis, thus unveiling their drug-likeness, good pharmacokinetics (PKs), and potential hypoglycemic properties. Altogether, our study established the preclinical efficacy of *T. garckeana* extract against inflammation, glycemic impairment, and oxidative stress, suggesting its future use for developing alternative therapies against diabetes complications.

## 2. Materials and Methods

### 2.1. Plant Collection, Extraction, and Fractionation

*Thespesia garckeana plant* was obtained from the Gombe State, Tula Village, Kaltungo Local Government Area (Latitude 9°48'51″N and Longitude11°18'32″E) Nigeria. Plant authentication was conducted at the Ebonyi State University, Nigeria, and a specimen voucher identification number was reported. The pulp was air-dried to a constant dry weight at 25°C for 2 weeks before been subjected to pulverization with the aid of a mechanical blending machine. Three hundred grams (300 g) of the pulverized sample was subjected to cold maceration in 1.5 L of methanol for 72 h. The resulting crude extract after concentration was further subjected to fractionation with diethyl ether, ethyl acetate, and n-butanol successively and the resulting fractions; diethyl ether (7.9% yield), ethyl acetate (3.10% yield), and n-butanol (2.45% yield) fractions were concentrated in a rotary vacuum evaporator under reduced pressure. The resulting diethyl ether fraction (dietl-eth_*T. garckeana*) which demonstrated the highest yield and phytochemical compositions in preliminary screening was used for subsequent analysis.

### 2.2. Analysis of Total Phenol and Flavonoid Contents

The total flavonoid content of the dietl-eth fraction of *T. garckeana* was determined according to the method of Chang et al. [33], while the total phenol content was evaluated using the Folin-Ciolcalteau's reagent as described by Singleton et al. [34]. Gallic acid (total phenols) and quercetin (total flavonoids) were used to prepare the calibration curves.

### 2.3. *In Vitro* Antioxidant Assays

The *in vitro* antioxidant effects of dietl-eth_*T. garckeana* were evaluated using the DPPH, FRAP, ABTS, and LPO assays. The DPPH assay was conducted based on the scavenge ability of the extract on DPPH radicals [35]. FRAP activity was assayed according to the method of Oyaizu [35]. The extract was incubated in K3[Fe(CN)6] supplemented phosphate buffer at 50°C for 20 min, after which 10% TCA was added. The resulting solution was centrifuged and color development in the presence of 0.1% ferric chloride was monitored at 700 nm. The thiobarbituric acid-reactive substance (TBARS) protocol described by Panjamurthy et al. [36] was employ for the LPO analysis, while the ABTS assay was done as reported by Re et al. [37].

### 2.4. *In Vitro* Hypoglycemic Assays

Standard analysis protocol for alpha-amylase inhibition described by Worthington [38] was employed for the *in vitro* hypoglycemic analysis of the extract. Briefly, the extract/acarbose (12.5 ~ 100 *μ*g/mL) mixed in an enzymatic porcine pancreatic solution was incubation with a starch solution for 30 min at 37°C, followed by the addition of 10 *μ*L of HCl (1 M) and iodine reagent for color development. In the analysis of glucose uptake by yeast cells, glucose solution (5 ~ 25 mM) was incubated (37°C for 10 min) with the extract. A yeast suspension was added to the reaction mixture and incubated for another 45-60 min, after which glucose concentration was estimated.

### 2.5. *In Vitro* Anti-Inflammatory Assays

The *in vitro* anti-inflammatory activities of the dietl-eth_*T. garckeana* were evaluated by using the human red blood cell (RBC and HRBC) membrane stabilization, inhibition of protein denaturation, and proteinase inhibitory assays. The protein denaturation inhibition was assayed as described of Mizushima and Kobayashi [39], while the method of Oyedepo and Femurewa [40] was used for the proteinase inhibition assay. In the HRBC assay, a 10% human RBC suspension was incubated with the extract solution at 50-55°C for 30 min [41]. The resulting solution was centrifuged at 2500 rpm for 5 min, and the reaction changes was monitored at 560 nm.

### 2.6. Analysis of Toxicity and Extract Tolerated Dose

The dietl-eth_*T. garckeana* was subjected to an acute toxicity study to evaluate its safety and maximum tolerated dose upon oral administration according to the protocol described by Lorke [42]. Briefly, a total of eighteen (18) rats were grouped into six (6) and administered with 10, 100, 1000, 1600, 2800, and 5000 mg/kg bw of dietl-eth_*T. garckeana* to groups I-VI, respectively. The extract was administered once orally, after which the rats were observed for any sign of adverse toxicity and mortality within 14 days' period.

### 2.7. Oral Glucose Tolerance Test (OGTT)

The OGTT was conducted according to the method described by Sisay et al. [43]. A total of twelve (12) overnight starved rats were grouped into 5 (3 rats per group) and treated with 2.0 mL/kg normal saline, 5 mg/kg glibenclimide, 150 mg/kg, and 300 mg/kg dietl-eth_*T. garckeana* for groups I-IV, respectively. After 30 minutes of treatment, 2 g/kg bw of glucose was orally administered to rats in each group. The blood glucose level was determined at 0 minutes (basal blood glucose level) and at intervals of 30 for a period of 2 hrs.

### 2.8. Analysis of *In Vivo* Antidiabetic Activities in Rats

The dietl-eth_*T. garckeana* was evaluated for the *in vivo* antidiabetic effects in albino rats. Experimental rats were acquired from the experimental rodent facility of AE-FUNAI, Nigeria. Animal experiments were consented by the ethical committee of the AE-FUNAI, Nigeria. Streptozotocin (STZ; 40 mg/kg) was intraperitoneally administered to overnight starved rats, and rats with FBS > 200 mg/kg were considered diabetic [44]. Rats were rationed into 4 groups and treated with the dietl-eth_*T. garckeana* at 150 (group 1) and 300 mg/kg (group 2), 2 mL/kg normal saline (group 3), and 200 mg/kg metformin (group 4). Healthy rats were assigned to the fifth group to serve as the control. Treatments were given on a daily basis for a period of 21 days via oral route. The fasting blood sugar concentration and animals' body weight were checked during the study period.

### 2.9. *In Vivo* Anti-Inflammatory Analysis (Xylene-Induced Ear Swelling Assay)

The dietl-eth_*T. garckeana* was evaluated for its *in vivo* anti-inflammatory effects in mice using a xylene-induced ear edema test [45, 46]. Healthy mice were divided into five (*n* = 5) groups and administered orally with saline, dietl-eth_*T. garckeana* (150 and 300 mg/kg BW), or 200 mg/kg aspirin for 7 days. On the eighth day, the posterior and anterior surfaces of the rat's right ear were scrubbed with 0.02 mL aliquot of xylene. Measurement of the ear thickness was done, and the ear inflammation volume was computed as the weight differences between the right and the left ears. Orbital blood sample was also collected and analyzed for the levels of white blood cell counts.

### 2.10. Collection and Processing of Samples

Experimental rats were euthanized under diethyl ether vapor for few minutes, and blood were obtained through cardiac puncture. The coagulated blood was centrifuged for 15 min (3000 × *g*) and the separated serum were stored at 4°C [47, 48]. The rat's pancreases and liver tissues were harvested, homogenized in phosphate buffer (0.1 M, pH 7.4, 1 : 10 *w*/*v*) and centrifuged at 10,000 × *g* for 10 min at 4°C.

### 2.11. Tissue Assays for Antioxidant Parameters

The *in vivo* antioxidant effects of the extract were evaluated in liver homogenate using the lipid peroxidation (LPO), superoxide dismutase (SOD), reduced glutathione (GSH), and catalase (CAT) assays. The LPO assay was conducted by measuring the tissue levels of TBARSs [49], while the modified colorimetric method of Kum-Tatt and Tan [50] was adopted for GSH assay. The activity of SOD was evaluated by using an established protocol [51]. The tissue homogenate was incubated with carbonate buffer (pH 10.2) in the presence of 0.3 mM adrenaline. The increase in absorbance at an interval of 30 s were monitored at 480 nm [51]. CAT activity was determined using a standard protocol [52]. The liver homogenate (0.1 mL) in phosphate buffer (0.01 M, pH 7.0) was incubated with 2 M hydrogen peroxide (H_2_O_2_) for 20 min. This was followed by the addition of a dichromate acetate reagent to terminate the reaction. The changes in absorbances were monitored at the wavelength of 620 nm and the activity of CAT was expressed as a unit of H_2_O_2_/mg protein [52].

### 2.12. Measurement of Insulin Levels

The Insulin ELISA kit (Calbiochem-Behring Corp, CA; catalog no. IN374S) was used for the analysis of pancreatic and serum insulin concentrations as per the manual instructions.

### 2.13. High-Performance Liquid Chromatography (HPLC)

The dietl-eth_T. garckeana was subjected to HPLC analysis according to the method described in our previous study [20]. A hundred milligram (100 mg) of dietl-eth_T. garckeana was dissolved in five millilitres (5 mL) of an HPLC grade methanol. The extract solution was filtered and run on the HPLC (Agilent Technologies 1200) with the following chromatographic conditions: stationary phase (Hypersil BDS C18), mobile phase (acetonitrile and 0.1% formic acid), column dimension of 250 mm × 4.0 mm, injection volume of 10 *μ*L, a flow rate of 0.6 mL/min, detector wavelength of 280 nm, and at gradient mode of elution.

### 2.14. Pharmacoinformatics Analysis

#### 2.14.1. Analysis of Drug-Likeness and ADMET-PK Properties

The most abundant compounds identified from the dietl-eth_*T. garckeana* were subjected to analysis of physicochemical properties, drug-likeness, PKs (ADMET), and medicinal chemistry using the ADMET-Lab, ADMETSar, and Swiss-ADME servers [53]. The human-intestinal absorption and permeability by blood-brain barrier (BBB) were modeled through the BOILED-EGG and support vector machine (SVM) tools [53].

#### 2.14.2. Analysis of Ligand Receptor Interactions Using Molecular Docking

The most abundant bioactive compound characterized from the extract was subjected to an analysis of ligand-receptor interactions using molecular docking. The crystal three-dimensional (3D) forms of the compounds were built by the Avogadro visualization tool (version 1.XX) [54], while the PDB files of the target receptors including alpha-amylase and alpha-glucosidase were downloaded from the Protein Data Bank. The mol2 files were transformed into PDB files using PyMOL software, while the PDB formats were transformed to PDBQT using AutoDock Vina [55]. Water molecules were detached, while hydrogen atoms in polar forms and Kollman charges were added to the compounds during predocking preparations [56, 57]. The version 8 of AutoDock Vina tool was used for the receptor-ligand docking as described in previous studies [56, 58, 59], while visualization was conducted using the PyMOL and Discovery studio tools [60].

### 2.15. Data Analysis

The GraphPad vers. 8.0 software was used for the statistical analysis of replicate data. The one-way type analysis of variance and Student's *t*-test were explored for statistical comparison between groups. Data are presented as the mean ± standard error of the mean (SEM), and statistical annotation of “^∗^,” “^∗∗^,” or “^∗∗∗^,” were used to represents the statistical differences corresponding to “*p* < 0.05,” “*p* < 0.001,” and “*p* < 0.001,” respectively.

## 3. Results and Discussion

### 3.1. *Thespesia garckeana* Fraction Exhibited Dose-Related *In Vitro* Antioxidant Activities

Plants, particularly those that are rich source of flavonoid and polyphenolic compounds, have been effective against various diseases including cancers and diabetes [61]. These phytochemicals are known to exhibit several biological activities including antioxidants, antimicrobial, anticancer, anti-inflammatory, and antidiabetic, etc. [62]. Interestingly, our results demonstrated that dietl-eth_*T. garckeana* contains total phenol contents of 613.65 ± 2.38 mg/100 g dry weight and total flavonoid contents of 152.83 ± 1.56 mg/100 g dry weight. The flavonoid and phenol contents reported in this study are higher than the total phenol (34.32 and 25.34 mg/100 g) and flavonoid (13.45 and 7.65 mg/100 g) contents previously reported for crude methanol and ethyl-acetate extracts of *T. garckeana* [28]. The presence of significant-high amounts of phenol and flavonoid contents in the dietl-eth_*T. garckeana* suggested the ability of the extract to scavenge free radicals, prevent oxidative stress and manage diabetes [62].

**Figure 1:**
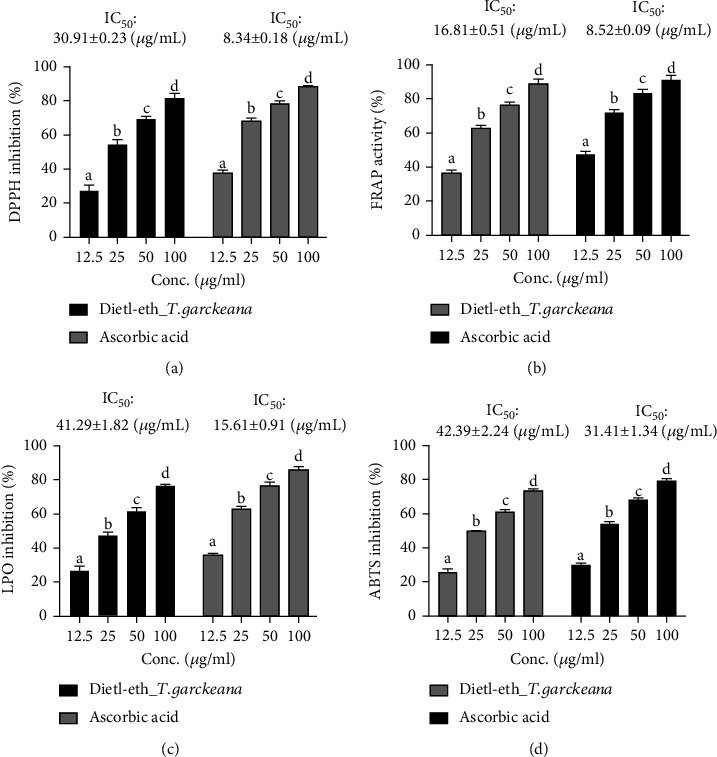
Diethyl-ether fraction of *Thespesia garckeana* (dietl-eth_*T. garckeana*) exhibited dose-related *in vitro* antioxidant activities. Bar graphs showing extract dose vs. inhibition effect of the dietl-eth_*T. garckeana* on (a) 2,2-diphenyl-1-picrylhydrazyl (DPPH) radicals, (b) ferric-reducing antioxidant power (FRAP), (c) lipid peroxidation (LPO), and (d) 2,2'-azino-bis (3-ethylbenzthiazoline-6-sulphonic acid (ABTS). Values are the mean ± SEM (*n* = 3). Different superscript letters indicate significant differences (*p* < 0.05) between the extract doses. IC_50_: half-maximal inhibitory concentration.

The formation of free radicals and oxidative stress play important roles implicated in the development of T2DM [12, 13]. Therefore, the role of antioxidants in preventing the formation of free radicals is crucial to the control of DM [61]. Our *in vitro* studies revealed significant and dose-related antioxidant activities of the dietl-eth_*T. garckeana* in four different *in vitro* models of antioxidant assays; DPPH, FRAP, LPO, and ABTS, with IC_50_ values of 30.91 ± 0.23 (Figure 1(a)), 16.81 ± 0.51 (Figure 1(b)), 41.29 ± 1.82 (Figure 1(c)), and 42.39 ± 2.24 *μ*g/mL (Figure 1(d)), respectively. Through the analysis of TBARS levels, we uncovered that the malonaldehyde (MDA) formation has been significantly compromised by dietl-eth_*T. garckeana*. In addition, the hydrogen donating properties of the extract as a means of radical scavenging has been demonstrated by the DPPH assay [63]. The reduction of Fe^3+^/Fe^2+^ as suggested by the FRAP assay also confirmed the scavenging ability of dietl-eth_*T. garckeana*, thereby transforming reactive radical element into a more stable product. Phenols and flavonoids were implicated in the free radical scavenging abilities and defensive properties of plants against various illnesses [64]. Consequently, the high flavonoid and phenolic contents of dietl-eth_*T. garckeana* may be appraised for the antioxidants activity of the extract.

### 3.2. *Thespesia garckeana* Fraction Exhibited Dose-Related *In Vitro* Anti-Inflammatory Activities

**Figure 2:**
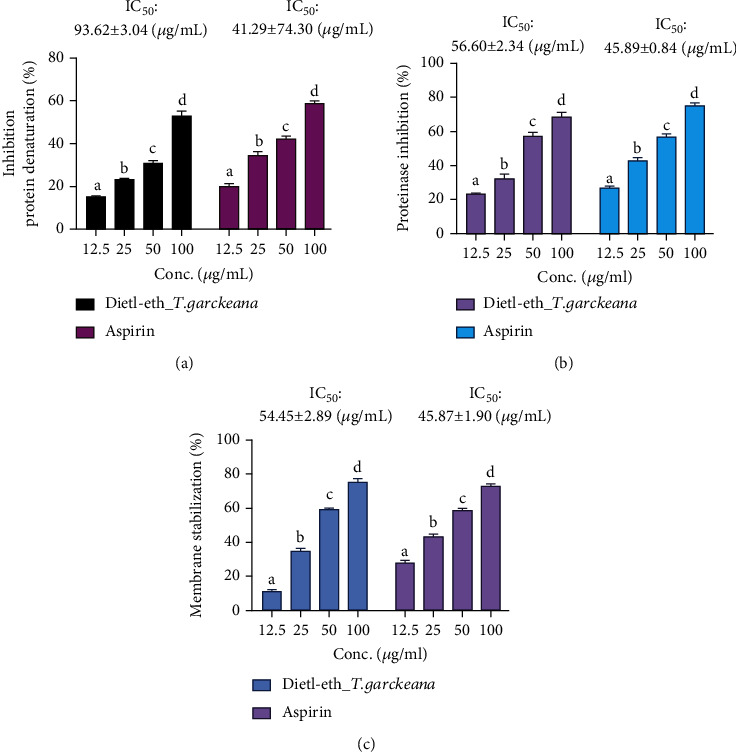
Diethyl-ether fraction of *Thespesia garckeana* (dietl-eth_*T. garckeana*) exhibited dose-related anti-inflammatory activities *in vitro*. Bar graphs showing extract dose vs. inhibition effect of the dietl-eth_*T. garckeana* on (a) inhibition of protein denaturation, (b) inhibition of proteinase activities, and (c) membrane stabilization. Values are the mean ± SEM (*n* = 3). IC_50_: half-maximal inhibitory concentration.

Inflammation, a complex physiological response to injury and infection, plays a pivotal role in the development of chronic disorders, including arthritis, asthma, atherosclerosis, cancer, and DM [14]. Interestingly, *T. garckeana* exhibited dose-related anti-inflammatory activities. Our *in vitro* anti-inflammatory studies using membrane stabilization, protein denaturation, and proteinase activities revealed the effectiveness of the extract with respective IC_50_ values of 54.45 ± 2.89, 93.62 ± 3.04, and 56.60 ± 2.34 *μ*g/mL (Figures 2(a)–2(c)). The erythrocytic membrane exhibits some similarities with the lysosomal membrane and its stabilization implies that the dietl-eth_*T. garckeana* may well stabilize lysosomal membranes [65]. This is important for restraining the inflammatory response by halting the lysosomal releases of active neutrophils, such as proteases and bacterial enzymes, which may induce tissue inflammation and injury upon extracellular release [66]. The dietl-eth_*T. garckeana* promoting membrane stabilization also suggests its potential for mitigating phospholipases release thereby preventing the generation and activities of inflammatory mediators [67].

Proteinase inhibitory activity was studied to further explain the anti-inflammatory mechanism of dietl-eth_*T. garckeana*. Neutrophils contains high levels of serine proteinase [65] that plays a vital tissue damaging role during inflammatory events [68] and inhibitors of this proteinase protect against the damage [69]. Interestingly, the dietl-eth_*T. garckeana* exhibited dose related antiproteinase activity with IC_50_ of 56.60 ± 2.34 *μ*g/mL. These results provide evidence for proteinase inhibition as an additional mechanism of the anti-inflammatory effect of dietl-eth_*T. garckeana*. Altogether, these data suggest that *T. garckeana* extract would be useful for averting inflammatory complications that could be associated with diabetes.

### 3.3. *Thespesia garckeana* Fraction Demonstrated Safety Profile in Acute Oral Toxicity Study

According to the data obtained from this study, dietl-eth_*T. garckeana* demonstrated a good safety profile with a 50%lethal dose (LD_50_) > 500 mg/kg bw and a safe dose of 1000 mg/kg bw (Table 1). No animal mortality was observed during the study. In addition, rats dosed with 10, 1000, and 1000 mg/kg bw were bereft of adverse health or physiological changes, suggesting the safety of the extract at doses ≤ 100 mg/kg bw. Conversely, rats treated with 1600, 2800, and 5000 mg/kg exhibited varying levels of adverse effects ranging from restlessness, hyperactivity, redness of the eyes, and profuse breathing. According to Hodge and Sterner [70], substances that demonstrate an LD_50_ of 5000 mg/kg in rats should be considered harmless substances. This result is in agreement with the findings of Iyojo et al. [71], who reported no mortality in rabbits administered different extracts of *T. garckeana* pulp at 5000 mg/kg. Altogether, dietl-eth_*T. garckeana* demonstrated high LD_50_ and is safe for use as an oral remedy at doses ≤ 100 mg/kg bw. Thus, the high safety of this plant upon oral exposure justifies the widespread use of this plant for treating various ailments by traditional healers in northern Nigeria.

### 3.4. *Thespesia garckeana* Fraction Exhibited Dose-Related *In Vitro* Hypoglycemic Activities

**Figure 3:**
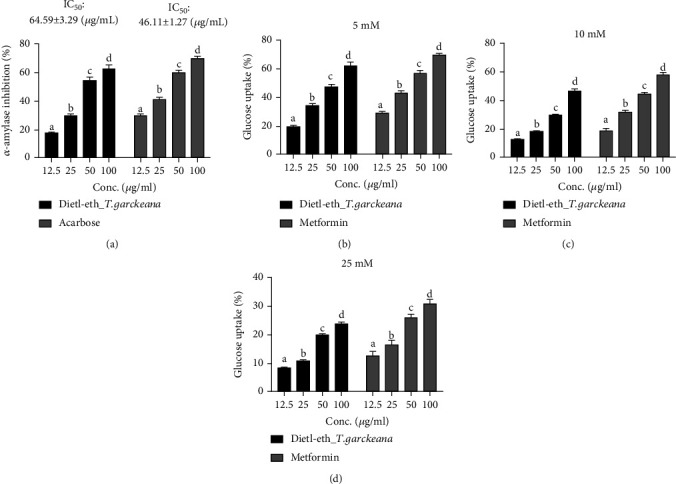
Diethyl-ether fraction of *Thespesia garckeana* (dietl-eth_*T. garckeana*) demonstrated *in vitro* dose-related hypoglycemic activities. Bar graphs showing extract dose vs. inhibitory effects of the dietl-eth_*T. garckeana* on (a) *α*-amylase inhibition, and yeast glucose uptake inhibition at (b) 5, (c) 10, and (d) 25 mM concentrations of glucose. Values are the mean ± SEM (*n* = 3). IC_50_: half-maximal inhibitory concentration.

The inhibition of carbohydrate-metabolizing enzymes and regulation of blood glucose levels are very critical to the management of DM [72]. The dietl-eth_*T. garckeana* also demonstrated a hypoglycemic effect via inhibition of *α*-amylase (IC_50_: 64.59 ± 3.29 *μ*g/mL) and enhanced glucose (5, 10, and 25 mM) uptake by yeast cells in a dose-related manner (Figure 3).

Inhibitors of *α*-amylase, a starch-catabolizing enzyme, are widely used as oral hypoglycemic agents for the regulation of sugar levels of T2DM patient [73]. However, most of these inhibitors are synthetic with of off-limit activity and associated side effects [74]. Consequently, the current results verify that the dietl-eth_*T. garckeana* exerts its hypoglycemic via the inhibition of carbohydrate-catabolizing enzymes, including the *α*-amylase. The inhibitory activities of the dietl-eth_*T. garckeana* on *α*-amylase activities suggests the attenuation of postprandial blood glucose increase by decreasing the carbohydrates flow into the bloodstream after carbohydrate intake [75].

Glucose is preferred by yeast as a primary source of fuel, and glucose absorption by yeast cells is simulate that occurring in the mammalian intestinal lumen; thus, yeast has become a commonly used model to study glucose absorption [76]. According to the present study, dietl-eth_*T. garckeana* significantly enhanced glucose uptake by yeast cells in a dose-related manner. This is important for the efficient utilization and control of glucose levels. Interestingly, a linear relationship between glucose concentration and rates of glucose uptake by the yeast cells was observed. This is in line with the finding of Keshala et al. [61] who reported a concentration-dependent increase in glucose uptake by yeast cells in the presence of plant extract. Collectively, the dietl-eth_*T. garckeana* exhibited dose-related *in vitro* hypoglycemic activities and, thus, could be regarded as a natural product with potential for the management of DM.

### 3.5. The dietl-eth_*T. garckeana* demonstrated hypoglycemic effect in Oral glucose tolerance test

**Figure 4:**
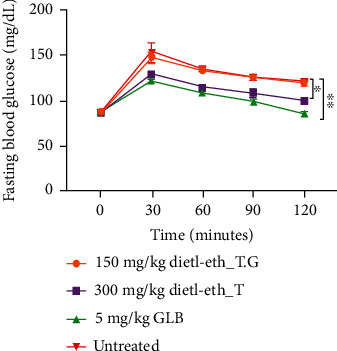
Effect of the diethyl-ether fraction of *Thespesia garckeana* (dietl-eth_*T. garckeana*) on blood glucose levels in oral glucose tolerance test (OGTT). Values are the mean ± SEM. ^∗^*p* < 0.05, ^∗∗^*p* < 0.01.

In the present study, the evaluation of dietl-eth_*T. garckeana* for possible antidiabetic effect was also conducted using the oral glucose tolerance test. There were initial increases in glucose levels at 30 minutes of glucose dosing after which progressive decreases were observed from 30 minutes to 2 hours in all the treatment as well as the control rats. However, significant (*p* < 0.05) decreases in the glucose levels of rats treated with the 300 mg/kg dietl-eth_*T. garckeana*, as well as the standard control group, were observed when compared with the nontreated glucose-loaded rats (Figure 4). Our result has shown that the extract can decrease postprandial blood glucose levels and improve peripheral glucose uptake and utilization in rats. The OGTT corroborate the effects of the plant extracts on insulin release by the B cells of the islets of Langerhans in diabetic rats. Postprandial elevated sugar level is with an increased risk of diabetes-associated secondary complications [77]. Therefore, the OGTT suggested the potential usefulness of dietl-eth_*T. garckeana* in T2DM subjects with insulin resistance prone to elevated postprandial sugar level [78].

### 3.6. The dietl-eth_*T. garckeana* Demonstrated Hypoglycemic Effect and Improved Insulin Secretion in STZ-Induced Diabetic Rats

**Figure 5:**
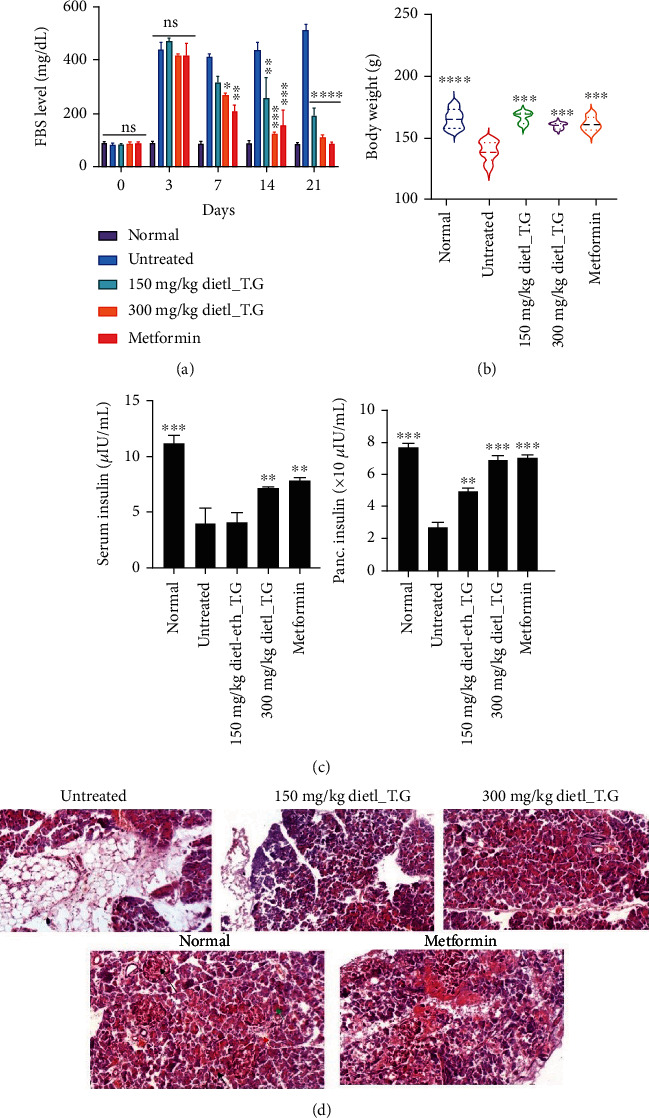
Diethyl-ether fraction of *Thespesia garckeana* (dietl-eth_*T. garckeana*) demonstrated a hypoglycemic effect and improved insulin secretion in STZ-induced diabetic rats. Bar graph showing the effects of the dietl-eth_*T. garckeana* on (a) fasting blood glucose, (b) body weight, and (c) pancreatic and insulin levels in diabetic rats. (d) Cross-section of the STZ-induced diabetic rat's pancreas administered dietl-eth_*T. garckeana.* Long arrow: islet of Langerhans; short arrow: interlobular septum; red arrow: pancreatic duct; green arrow: acini. Values are the mean ± SEM. “^∗^*p* < 0.05,” “^∗∗^*p* < 0.01,” “^∗∗∗^*p* < 0.001,” and “^∗∗∗∗^*p* < 0.0001”.

Previous experimental studies have proved the therapeutic efficacy of medicinal plants in animal models of diabetes, inflammation, and oxidative stress-associated diseases [79–81]. The *in vivo* antidiabetic effects of the dietl-eth_*T. garckeana* was evaluated in STZ-induced diabetic rats. Interestingly, our *in vitro* findings corroborated with the data generated *in viv*o. We found that treatment of the STZ-induced diabetic rats with dietl-eth_*T. garckeana* caused significant and progressive decreases in the fasting blood sugar (FBS) levels in a dose-related manner (Figure 5(a), Table 2) and prevented the body weight loss (Figure 5(b), Table 3), while diabetic untreated rats exhibited progressive increases in FBS levels and body weight loss. At the end of the treatment duration, the extract at 150 and 300 mg/kg demonstrated 62.67% and 78.88% hypoglycemic effects, respectively, while metformin demonstrated higher hypoglycemic activity of 84.25%. Thus, the gradual decreases in FBS and improvement of body weight recorded in the *T. garckeana*-receiving animal when compared with the untreated diabetic rats presaged the ameliorative effects of the fraction on experimentally induced diabetes.

Under a physiological condition, the pancreatic cells regulate blood glucose levels by regulating *β* cells of Islets of Langerhans's activity through insulin secretions. Therefore, the hyperglycemia induced by STZ may be attributed to the impairment of insulin release as a consequence of the destroyed *β* cells of Islets of Langerhans in the pancreas [82]. Indeed, we found that the STZ-induced diabetic nontreated rats exhibited significant (*p* < 0.05) decreases in the pancreatic and serum (Figure 5(c)) insulin levels as well as histological distortion of the pancreas (Figure 5(d)) when compared with the control rats, while administrations of the dietl-eth_*T. garckeana* significantly (*p* < 0.01) increased the serum and pancreatic insulin levels of the 300 mg/kg-treated diabetic rats only. The serum insulin level in 150 mg/kg-treated rats was not significantly (*p* > 0.05) different from diabetic nontreated rats. Furthermore, the pancreatic section of the STZ-induced diabetic nontreated rats shows a pancreatic architecture with loose connective tissue. The parenchymatous portion of acini and islet are distorted and most enriched by adipose tissue (Figure 5(d)). However, in a similar architecture to the normal control rats, the pancreatic section of rats treated with metformin, and the 300 mg/kg extract receiving rats show the well-preserved pancreatic architectures, comprising lobules of exocrine acini separated by thin fibrous septa. Normal islets of Langerhans are seen, and there were no features of significant inflammation or damage seen. These ameliorative effects of the extract on pancreatic architecture, activity, and insulin secretion might have promoted the effective glucose uptake and utilization [83] and thus restored the glycemic status of the rats. Altogether, this study provides preclinical evidence supporting the potential therapeutic benefits of dietl-eth_*T. garckeana* in stimulating insulin secretion and attenuating hyperglycemia in strepzotocin-induced diabetic rats.

### 3.7. The dietl-eth_*T. garckeana* Exhibited Antioxidant Activities in Rats with STZ-Induced Diabetes

**Figure 6:**
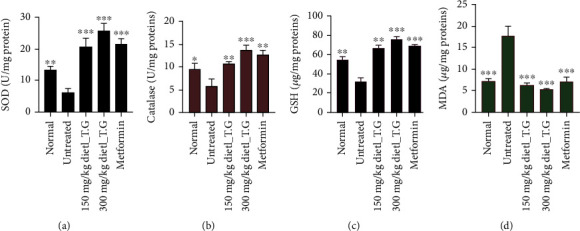
The diethyl-ether fraction of *Thespesia* (dietl-eth_*T.*) *garckeana* demonstrated antioxidant activities in diabetic rats. Bar graphs show the effect of the dietl-eth_*T. garckeana* on levels of (a) superoxide dismutase (SOD), (b) catalase (CAT), (c) reduced glutathione (GSH), (d), and malonaldehyde (MDA) in diabetic rats. Data = mean ± SD, *n* = 3. “^∗∗∗^*p* < 0.001,” “^∗∗^*p* < 0.01,” and “^∗^*p* < 0.05”.

Inflammation and oxidative stress contribute to the development of diabetic complications such as hypertension, retinopathies, nephropathies, and neuropathies [19, 20]. The generation levels of ROS are controlled by the levels endogenous antioxidant including CAT, GSH, and SOD [84, 85]. In the present study, analysis of liver biochemical parameters of oxidative stress in rats with STZ-induced diabetes revealed significant decreases in the liver activities of antioxidant enzymes including SOD (*p* < 0.01; Figure 6(a)), CAT (*p* < 0.05; Figure 6(b)), and GSH (*p* < 0.01; Figure 6(c)) and increased MDA levels (*p* < 0.001; Figure 6(d)) in STZ-induced diabetic rats compared to the respective normal controls.

The liver is a central detoxification organ of the body and plays a vital role in regulating glucose homeostasis [86]. As a group of insulin-sensitive tissues, the liver is among the primary organs highly susceptible to the effects of hyperglycemia-provoked oxidative stress, which may impair liver integrity [87]. Hyperglycemia, mainly caused by insulin resistance, induces the generation of free radicals by the activated Kupffer cells (phagocytic hepatic macrophages) that help in maintaining the integrity of liver cells [88]. However, these cells are highly susceptible to the effects of the free radicals generated by their own immune reactions and the surrounding cells [89]. The excessive ROS production results in irreversible oxidative alterations of macromolecules including the carbohydrates, lipids, and proteins [90], thereby leading to increased oxidative stress and triggering the cascade of inflammatory events that activates the transcription of proapoptotic genes and damages hepatocytes [91, 92].

Therefore, the reduced SOD and CAT activities in the liver of STZ-induced diabetic rats may be associated with the free radical generations which inturm decreases the activities of these enzymes. GSH is a cellular defense antioxidant molecule that protects against the progressive destruction of the ß cell [93]. The increases in free radicals' productions in the diabetic rat result in oxidative damage to membrane lipids and proteins and eventually causes a decrease in the levels of GSH, CAT, and SOD as recorded in rats with STZ-induced diabetes. However, our results revealed that treatment with the dietl-eth_*T. garckeana* attenuated depleted levels of GSH, CAT, and SOD and decreased the LPO. These observations validate the potential of dietl-eth_*T. garckeana* in preventing free radical generation and maintaining the antioxidant status of diabetic rats [93]. This finding corroborated with literature on oxidative stress-alleviating properties of plant extracts in hyperglycemic rodent [94]. Our findings, therefore, revealed the *T. garckeana*'s ability to ameliorate oxidative impairment and restore antioxidant status of rats in hyperglycemic condition.

### 3.8. The dietl-eth_*T. garckeana* Demonstrated *In Vivo* Anti-Inflammatory Activities in Xylene-Induced Ear Swelling of Mice

An *in vivo* analysis of the anti-inflammatory properties using a xylene-induced ear swelling model in mice revealed that the dietl-eth_*T. garckeana* significantly (*p* < 0.001) prevented xylene-induced ear swelling compared to the untreated control. The anti-inflammatory effect demonstrated by the extract was accompanied by a significant (*p* < 0.001) decrease in WBC counts of treated mice compared to the untreated control (Table 4). Collectively, our results demonstrated that the dietl-eth_*T. garckeana* exhibited an anti-inflammatory effect *in vivo* in addition to its *in vitro* anti-inflammatory effects.

### 3.9. HPLC Characterization of the dietl-eth_*T. garckeana*

**Figure 7:**
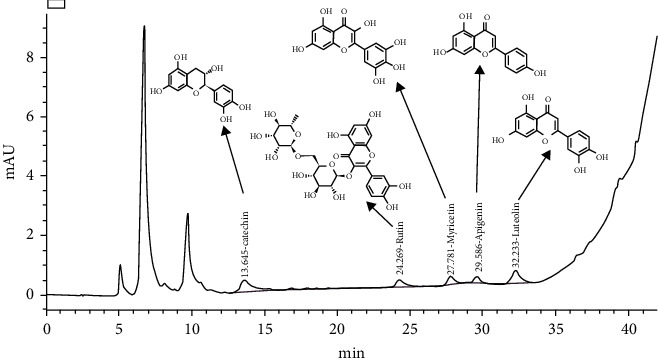
High-performance liquid chromatography (HPLC) chromatogram and chemical structures of bioactive compounds identified from the diethyl-ether fraction of *Thespesia garckeana.*

To characterized the dietl-eth_*T. garckeana* we conducted an HPLC analysis (Figure 7) and identified the presence of catechin (6.81e-1 ppm), rutin (8.46 e-1 ppm), myricetin, apigenin (4.019 e-1 ppm), and luteolin (15.09 ppm) with respective retention times (RTs) of 13.64, 24.269, 27.781, 29.58, and 32.23 min. HPLC chromatograms and chemical structures of the compounds are displayed in Figure 7. Luteolin appeared to be the most abundant compound in the dietl-eth_*T. garckeana*, and thus, we evaluated its prospective for targeting glucose metabolizing enzymes and an inflammatory mediator.

### 3.10. Drug-ADMET and Likeness Modeling of Compounds Identified from the dietl-eth_*T. garckeana*

**Figure 8:**
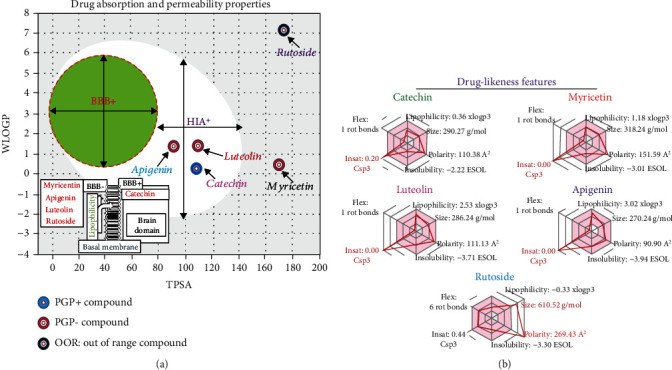
Drug-likeness and permeability simulation of compounds identified from the dietl-eth_*T. garckeana* (a) absorption and permeability and (b) drug-likeness modeling of compounds from the diethyl-ether fraction of *Thespesia garckeana*.

The goal of modern drug discovery and development is to identify a drug candidate with desirable properties within the shortest possible period of time and to avoid time- and cost-consuming approaches which in most cases produces disappointing outcome in the clinics [95, 96]. Hence, drug-likeness and PK analysis is considered an important aspect of the modern drug discovery and development. Our modeling analysis of the drug-PK, and drug-likeness revealed that apigenin, myricetin, luteolin, and catechin identified from the dietl-eth_*T. garckeana* (Figures 8(a) and 8(b)) were potential drug-like molecules. These compounds passed the drug absorbability test and have desirable bioavailability attribute. P-glycoproteins (P-gp) are responsible for propelling compounds and drugs out of the cells [97]. The identified compounds from the dietl-eth_*T. garckeana* are nonsubstrate or inhibitors of Pgp, thus suggesting their stability and optimal drug delivery [98]. This was also evident by their absorption and permeability record. The high volume of distribution and low binding of plasma protein by the drug further ascertain the good bioavailability and hinted at the potential good therapeutic index of the compounds but were non-BBB permeant (except for catechin) (Figure 8(b), Table 5). Cytochrome P450 (CYP450) is a heme containing enzyme family that play central metabolic role on exogenous and endogenous substances [99]. Hence, impeding the activities of these enzyme isoforms may lead to deficient drug metabolism and toxic drug accumulation. Providentially, our data indicated that among the 5 isoforms analyzed, the compounds were nonsubstrate and had inhibitor tendencies for cytochrome P450 1A2 (CYP1A2) and CYP3A4. However, they were nonsubstrate nor inhibitors of CYP2C9, CYP2D6, and CYP2C19. Notwithstanding, luteolin demonstrated its ability to be a substrate for P450 CYP3A4 and CYPC19, while catechin demonstrated its ability to be a substrate for CYP2D6 (Table 5). The presence of these isoforms in the liver and intestines indicates that these organs are sites of clearance of the compounds. Among the five compounds, luteolin and catechin demonstrated the best and most similar half-lives of 0.745 and 0.720 h, high clearance rates of 1.919 and 1.914 mL/min/kg, and a high safety profile with LD_50_ values of 737.444 and 860.605 mg/kg, respectively. Interestingly, luteolin and catechin demonstrated nontoxic attributes; nonhuman ether go-go-related gene (hERG) blockers, nonhepatotoxic, and were nonirritants in Skinsen assays. Collectively, our analysis revealed that among the five compounds identified, luteolin and catechin exhibited the best drug-PK and drug-likeness characteristic and, thus, were used for receptor-ligand simulation analysis.

### 3.11. Receptor-Ligand Simulation Analysis Revealed Luteolin's Properties for Targeting Glucose-Metabolizing Enzymes and an Inflammatory Mediator

**Figure 9:**
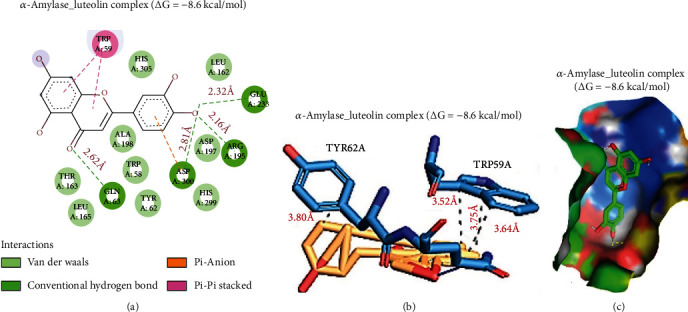
Docking of luteolin with *α*-amylase. (a) 2-Dimensional depiction of the luteolin complex with *α*-amylase, (b) 3-dimensional (3D) view of the hydrophobic interactions in the complex, and (c) surface representation of luteolin fitted within the binding cavity of the target.

Molecular docking is an innovative and widely approved strategies for mimicking a small-molecule interaction with a target receptor/protein [57, 100, 101]. It provides qualitative and quantitative estimation of the affinity between a compound and the corresponding protein/receptor [102]. It also gives a preamble insight into mechanistic aspect of the compound and its behavior when in contact with the corresponding target [103–107]. Docking analysis in the present study revealed that luteolin docked efficiently to the substrate interaction domain of *α*-amylase with a binding affinity of –8.6 kcal/mol. The complex was bound by four hydrogen bonds to Gln63 (2.62 Å), Asp300 (2.81 Å), Arg195 (2.16 Å), and Glu233 (2.32 Å); pi-pi stacked (Trp59), pi-anion (Asp300), and several van der Waals forces including His305, Leu162, Asp197, His299, Tyr62, Trp58, Ala198, Thr163, and Leu165 (Figure 9). In addition, the complex was bound by four hydrophobic contacts with Thr62A and TRP59A with interaction distances of 3.80, 3.52, 3.75, and 3.64 Å.

**Figure 10:**
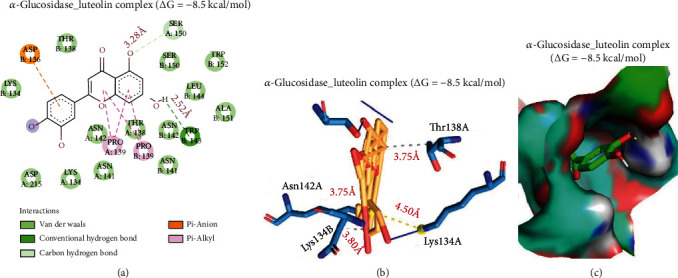
Docking of luteolin with *α*-glucosidase. (a) 2-Dimensional depiction of the luteolin complex with *α*-glucosidase, (b) 3-dimensional image of the hydrophobic links in the complex, and (c) surface representation of luteolin fitted within the binding cavity of the target.

Luteolin interacted with glucosidase by –8.5 kcal/mol binding efficacy. Luteolin was interposed to the cavity of glucosidase mainly by hydrogen bonds with Ser150 and TRP143 residues of glucosidase domain in respective proximity of 3.28 and 2.52 Å. The luteolin-glucosidase complex stabilization was also achieved by a pi-anion interaction with Asp136, two alkyl bonding with Pro139A and Pro139B, and several van der Waals forces, including Lys134A, Asp215, Lys134B, Asn141, Asn142A, Thr138, Asn142B, Ala151, Leu144, Ser150, and Trp152, molded at the luteolin backbone. In addition, there were three hydrophobic contacts with Thr138A (3.75 Å), Asn142A (3.75 Å), and Lys134B (3.80 Å), and the carboxylate group of luteolin formed a salt bridge interaction with the binding domain of alpha-glucosidase (Figure 10).

**Figure 11:**
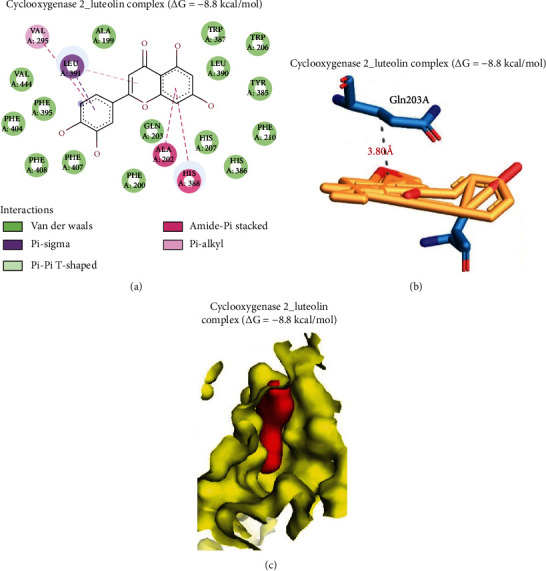
Molecular docking of luteolin with cyclooxygenase. (a) A 2-dimensional (2D) image of the luteolin complex with cyclooxygenase, (b) 3-dimensional (3D) image of the hydrophobic interactions in the complex, and (c) surface representation of luteolin fitted within the binding cavity of the target.

The luteolin-cyclooxygenase complex was stabilized by several pi interactions, including pi-alkyl (Val295), pi-sigma (Leu391), amide-pi-stacked (Ala202), and pi-pi T shape (His388), yielding a high ligand-binding affinity of –8.8 kcal/mol. In total, 13 van der Waals forces with Val444, Phe404, Phe395, Phe407, Phe200, Gln203, His207, His386, Phe210, Tyr385, Leu390, Trp387, and Thr206 residues of the cyclooxygenases were found around the luteolin backbone. In addition, hydrophobic contact of Gln203A with proximity of 3.80 Å was found in the complex (Figure 11). Overall, data presented in this study provides some scientific affirmation based on preclinical model of the anti-inflammatory, hypoglycemic, and antioxidant properties of the *T. garckeana* extract. This hinted at its potentiality for exploration in the development of alternative therapies for the management and possibly treatment of diabetes complication

## 4. Conclusions

The present study provides experimental evidence of the therapeutic efficacy of the diethyl-ether fraction of *Thespesia garckeana* for treating diabetes. The extract not only enhanced the activities of antioxidant enzymes but also inhibited inflammatory responses, and demonstrated *in vivo* antidiabetic effects in experimental models. The compound most abundant (luteolin) in the fraction demonstrated good drug-PK and drug-likeness and prospective for the targeting of glucose-catabolizing enzymes. Thus, the present study provides preclinical insights into the bioactive constituents of *T. garckeana*, its anti-inflammatory and antioxidant effects, and its potential for treating diabetes.

## Figures and Tables

**Table 1:** Acute oral toxicity profiles of diethyl-ether fraction of *Thespesia garckeana* in rats.

dietl-eth_*T. garckeana* (mg/kg)	Mortality	Adverse effect	Two weeks' posttreatment observation (loss of weight, sign of toxicity)
10	0/3	Nil	Nil
100	0/3	Nil	Nil
1000	0/3	Nil (MTD)	Nil
1600	0/3	Restlessness (10-15 mins)	Nil
2900	0/3	Rubbing of the mouth on the wall of the cage (about 20 mins); restlessness (about 20 mins)	Nil
5000	0/3	Rubing of cages (20-30 mins), redness of the eye (30 mins), restlessness (30 mins), fur erection (30 mins), profuse breathing (24 h), weakness (only in day 2)	Nil

MTD: maximum tolerated dose.

**Table 2:** Effect of diethyl-ether fraction of *Thespesia garckeana* on blood glucose levels in STZ-induced diabetic rats.

Groups	0	3	7	14	21	Glucose reduction (%)
Normal	89.66 ± 1.76	88.06 ± 3.60	87.06 ± 4.58	90.66 ± 3.71	85.05 ± 1.15	—
Untreated	84.05 ± 3.00	442.50 ± 17.50	415.5 ± 5.500	441.50 ± 18.50	518.50 ± 12.50	—
150 mg/kg Dietl_T.G	82.03 ± 3.01	474.50 ± 6.50	319.50 ± 14.50	258.5 ± 54.5	193.55 ± 19.05	62.67
300 mg/kg Dietl_T.G	87.00 ± 3.00	419.50 ± 2.50	269.60 ± 4.40	125.70 ± 4.30	109.46 ± 5.87	78.88
Metformin	86.00 ± 2.08	433.33 ± 24.03	210.66 ± 15.76	169.33 ± 35.25	81.66 ± 4.25	84.25

Dietl_T.G: diethyl-ether fraction of *Thespesia garckeana.*

**Table 3:** Effect of diethyl-ether fraction of *Thespesia garckeana* on body weight gain in STZ-induced diabetic rats.

Groups	0	3	7	14	21	Weight gain (%)
Normal	157.05 ± 2.00	159.01 ± 1.54	165.22 ± 1.31	169.80 ± 1.29	176.53 ± 2.64	11.05 ± 0.19
Untreated	146.85 ± 3.86	146.03 ± 9.38	138.83 ± 8.60	136.17 ± 4.05	126.79 ± 3.30	−15.82 ± 0.03
150 mg/kg Dietl_T.G	163.26 ± 9.92	160.26 ± 8.93	169.25 ± 10.64	169.60 ± 8.61	169.33 ± 5.30	4.01 ± 2.85
300 mg/kg Dietl_T.G	158.27 ± 0.72	156.42 ± 1.34	160.63 ± 0.51	161.54 ± 0.68	162.19 ± 1.08	2.40 ± 1.09
Metformin	160.20 ± 0.88	154.37 ± 2.84	158.42 ± 4.19	163.93 ± 3.81	169.60 ± 1.49	5.53 ± 0.31

Dietl_T.G: diethyl-ether fraction of *Thespesia garckeana*.

**Table 4:** *In vitro* anti-inflammatory effects of the diethyl-ether fraction of *Thespesia* (dietl-eth_*T*.) *garckeana.*

Treatment	Dose (mg/kg BW)	Swelling rate	WBCs (×10^9^)
dietl-eth_*T. garckeana*	150	16.71 ± 1.19^∗∗∗^	106.00 ± 1.73^∗∗∗^
dietl-eth_*T. garckeana*	300	10.41 ± 1.56^∗∗∗^	98.00 ± 1.1561^∗∗∗^
Aspirin	200	13.67 ± 1.20^∗∗∗^	101.00 ± 3.46^∗∗∗^
Untreated control	2.5 mL/normal saline	77.33 ± 1.76	178.3.46 ± 3.46
Normal control	Normal control	—	106.00 ± 2.31^∗∗∗^

Values are the mean ± SD (*n* = 3). BW: body weight; WBCs: white blood cells; ^∗∗∗^ *p* < 0.001.

**Table 5:** Pharmacokinetic_drug-likeness characteristic of the compounds from the diethyl-ether fraction of *Thespesia garckeana.*

	Property	Rutin	Myricetin	Apigenin	Luteolin	Catechin
Physicochemical	Molecular weight	610.521	318.237	270.24	286.239	290.271
	Log*p*	-1.687	1.694	2.57	2.282	1.546
	HB acceptor	16	8	5	6	6
	HB donor	10	6	3	4	5
	TPSA	269.43	151.59	90.9	111.13	110.38
	LogS (solubility)	315.283 *μ*g/mL	191.757 *μ*g/mL	68.667 *μ*g/mL	90.101 *μ*g/mL	273.403 *μ*g/mL
	LogD7.4	0.992	-0.068	0.487	0.302	0.115
	Log*p*	-1.687	1.694	2.577	2.282	1.546
Absorption	Papp (colorectal adenocarcinoma permeability)	-6.606 cm/s	-6.63 cm/s	-4.985 cm/s	-5.123 cm/s	-4.637 cm/s
	P-glycoprotein-inhibitor	-(0.365)	---(0.208)	-(0.328)	-(0.366)	---(0.223)
	P-glycoprotein-substrate	---(0.168)	---(0.064)	---(0.023)	---(0.038)	---(0.045)
	Intestinal absorption (HIA)	---(0.21)	+(0.438)	+(0.531)	-(0.438)	-(0.401)
	Bioavailability	+(0.572)	+(0.557)	+(0.542)	+(0.557)	-(0.487)
Distribution	Plasma protein binding (PPB)	76.65%	76.595%	90.031%	91.796%	93.86%
	VD (L/kg)	-1.052	-1.391	-0.578	-0.934	-0.67
	*Blood brain barrier*	---(0.018)	-(0.369)	-(0.464)	-(0.464)	+++(0.708)
Metabolism	CYP-1A2 inhibitor	---(0.197)	+++(0.968)	+++(0.987)	+++(0.968)	---(0.034)
	CYP-1A2 subst.	---(0.258)	-(0.376))	-(0.408)	-(0.412)	-(0.368)
	CYP-3A4 inhibitor	+(0.518)	-(0.459)	+++(0.931)	++(0.867)	---(0.221)
	CYP-3A4 subst.	-(0.418)	-(0.33)	-(0.37)	-(0.328)	-(0.398)
	CYP-2C9 inhibitor	---(0.199)	+(0.656)	-(0.12)	---(0.071)	---(0.028)
	CYP-2C9 subst.	---(0.298)	+(0.557)	+(0.631)	-(0.496)	-(0.049)
	CYP-2C19 inhibitor	---(0.072)	---(0.068)	---(0.124)	---(0.124)	-(0.431)
	CYP-2C19 subst.	-(0.49)	-(0.345)	-(0.412)	+(0.542)	-(0.476)
	CYP-2D6 inhibitor	---(0.29)	-(0.318)	+(0.611)	-(0.463)	-(0.367)
	CYP-2D6 subst.	---(0.218)	-(0.18)	-(0.488)	-(0.401)	+(0.523)
Elimination	T 1/2 (half-life time)	2.138 h	0.915 h	1.331 h	0.745 h	0.720 h
	Clearance rate (mL/min/kg)	0.641	1.709	1.885	1.919	1.914
Toxicity/alert	hERG blocker	+(0.626)	-(0.353)	+(0.598)	-(0.436)	-(0.371)
	(hepatotoxicity (H-HT)	---(0.154)	-(0.332)	+(0.6)	+(0.592)	-(0.466)
	SkinSen	---(0.235)	---(0.278)	---(0.264)	---(0.278)	-(0.329)
	50% acute toxicity (LD_50_ mg/kg)	419.47	648.262	858.518	737.444	860.605

TPSA: topological polar surface area; CYP: cytochrome P450; hERG: human ether-a-go-go-related gene.

## Data Availability

All data used in this study will be made available upon reasonable request.
